# Health Equity, Hispanic/Latinx People and Truthfulness in Communicating the Role of the Advanced Practice Nurse

**DOI:** 10.1177/15404153241288519

**Published:** 2024-10-03

**Authors:** Rubi Alva de Hickson

**Affiliations:** Critical Care Nurse Practitioner

Having an accurate translation of the terms “advanced practice nurse” (APN) and “advanced practice registered nurse” (APRN) and their four advanced specialties, i.e., certified registered nurse anesthetist (CRNA), certified nurse midwife (CNM), clinical nurse specialist (CNS), and nurse practitioner (NP), is essential to health equity. Studies have found that misrepresenting NPs as specialized nurses rather than advanced practice NPs can be detrimental to Hispanic/Latinx patients (Escobedo et al., 2023). The literature highlights the importance of transparency and truthfulness in communicating the role of the advanced practice nurse, specifically the NP*,* particularly when dealing with the Hispanic/Latino community. By adhering to effective communication strategies and respecting cultural constructs, NPs can establish more robust and meaningful relationships with their patients, thereby improving healthcare experiences.

Translating the term “nurse practitioner” into Spanish affects the diverse Hispanic/Latinx community, which is comprised of individuals from various countries and cultures. Even though Hispanics/Latinos may be seen as a homogeneous group, they are, in fact, a diverse group of “many races, cultures, nationalities, language dialects, acculturation, educational attainment, migration histories, and economic levels” (Massett et al., 2021 p. 109). According to the U.S. Department of Health and Human Services Office of Minority Health (OMH), the profile of Hispanic/Latino Americans is “any person of Cuban, Mexican, Puerto Rican, South or Central America, or other Spanish culture or origin, regardless of race” (OMH, 2023).

Madeleine Leininger's theory of culture care diversity and universality inspires us to “generate a wealth of new and reaffirmed transcultural knowledge to care for immigrants, refugees, and many other minorities or majority cultures” (Fawcett, 2002, p. 134). It is imperative to have culturally based care that contributes to healing (health), well-being, and helping clients (Fawcett, 2002). Leininger's theory has several tenets that help guide the accurate translation of the term “nurse practitioner.” Understanding Hispanic/Latinx culture from the people's perspective, as well as the translation and how it influences that culture, is crucial. A significant tenet is understanding diversity and commonalities within a culture, which involves “expression, meanings, patterns, and practices” (Wehbe-Alamah, 2015, p. 306). Another important tenet is understanding the worldview and social structure factors that influence culture, such as “technology, religions (including spirituality and philosophy), kinship (family ties), cultural values, beliefs and lifeways, political and legal factors, economic and educational factors” (Wehbe-Alamah, 2015, p. 307). Finally, a third important tenet is understanding the difference between “the professional (etic) and the generic (emic) traditional, indigenous, or “folk” (Wehbe-Alamah, 2015, p. 307), which can be used to find the gap in translation.

Identifying the most accurate translation of “nurse practitioner” to Spanish is not just a matter of semantics. The incorrect translation of the nurse practitioner role could lead to significant misunderstandings among Hispanic/Latinx patients. Many patients may choose to wait to see a physician, believing that they are being seen by a “specialized nurse” rather than a provider. The words found in the umbrella term for “advanced practice registered nurse” (APRN) are not specific to the term nurse practitioner: “*enfermera (o) de práctica avanzada”*. Found in the literature when addressing NPs in Spanish are the words “*enfermera (o) practicante (o),*” or “*enfermera (o) practicante de avanzada.”*

Based on existing literature and social translations, it is essential to translate the term “nurse practitioner” accurately. One of the largest American Spanish-language broadcast television networks is Telemundo, which reaches 57.23% of all households in the United States. When addressing nurse NPs in California, “Telemundo” translates as “*enfermeras practicantes*” (telemundosareadeleabahia.com; telemundo52.com). Cigna Health, an insurance company with 19.5 million members by the end of 2022, translates the NP as “*enfermera practicante*” (https://www.cigna.com/es-us/knowledge-center/hw/enfermera-practicante-ps2031). CVS pharmacy, which has 9,564 CVS pharmacy locations in the United States as of July 10, 2023, translates the nurse practitioner as “*enfermera practicante*” (www.es.cvs.com).

When translating the term “*enfermera (o) practicante*” from Spanish to English, it's important to note that the direct translation “nurse practicing” does not accurately convey the concept of a practitioner. To accurately convey the role of a practitioner, the term “*avanzada*” must be added. Therefore, “*enfermera (o) practicante avanzada*” correctly translates to “nurse practitioner (provider role)” as opposed to simply a “nurse practicing”.

The **California** nursing community has expressed concerns about California's law AB 890 and its translation of the term “nurse practitioner” into Spanish. To ensure effective communication within the Hispanic/Latinx community and to promote health equity, it is crucial to establish the correct translation of this term. The translation of the “advanced practice nurse” and “advanced practice registered nurse” is “*enfermera (o) de práctica avanzada*.” Demonstrating the literal and social translation of the word “nurse practitioner,” the most accurate translation is “*enfermera (o) practicante avanzada*.” These accurate translations are crucial for compliance with legislation that requires advanced practice registered nurses and nurse practitioners to identify themselves to the public in Spanish. By using accurate translations, the healthcare community can better understand, collaborate, and connect which ultimately benefits everyone and moves us toward achieving health equity.
